# Analysis of bacterial and fungal communities in continuous-cropping ramie (*Boehmeria nivea* L. Gaud) fields in different areas in China

**DOI:** 10.1038/s41598-020-58608-0

**Published:** 2020-02-24

**Authors:** Yanzhou Wang, Xiaomin Xu, Touming Liu, Hongwu Wang, Yan Yang, Xiaorong Chen, Siyuan Zhu

**Affiliations:** 1 Institute of bast fiber crops, Chinese Academy of Agricultrial Sciences, Changsha Hunan, 410205 P.R. China; 2 Xianning Agriculture Academy of sciences, Hubei, China; 3 Dazhou Agriculture Academy of sciences, Sichuan, China; 4 Yichun Institute of Agricultural Sciences, Jiangxi, China

**Keywords:** Microbial ecology, Microbiome

## Abstract

Ramie (*Boehmeria nivea* L. Gaud) suffers from long-term continuous cropping. Here, using Illumina high-throughput sequencing technology, we aimed to identify bacteria and fungi associated with continuous cropping in ramie fields in Yuanjiang, Xianning, Sichuan, and Jiangxi. The rarefaction results showed that Jiangxi had significantly lower bacterial α-diversity than that of the other areas. Firmicutes, Proteobacteria, and Acidobacteria were the dominant bacterial phyla, and Ascomycota, Basidiomycota, and Zygomycota were the dominant fungal phyla. In Jiangxi, Firmicutes accounted for 79.03% of all valid reads, which could have significant decreased microbial diversity and negative effects of continuous ramie cropping. We used traditional methods to examine soil nutrients. Sichuan had a relatively high pH and available P and K, but low total N; opposite findings were recorded in Jiangxi. The redundancy analysis revealed that the urease activity, PH, available K, and total N significantly correlated with bacterial community abundance, whereas only total N significantly correlated with fungal community abundance (P < 0.01). Overall, the effect of soil environmental factors on the bacterial diversity of continuous ramie cropping was greater than that on fungal diversity. In the future, we will focus on the effect of rhizosphere bacteria to solve the obstacle in continuous ramie cropping.

## Introduction

In continuous cropping, the same or a similar species is planted continuously in the same field^1^. Generally, long-term continuous cropping decreases crop yield and quality^2–4^, alters soil microbial communities^5,6^, affects soil biochemical properties^7,8^, and enriches soil-borne plant pathogens in soil^9,10^. Soil microorganims are not only an important part of soil, but also the main drivers of soil nutrient cycling^11^. Soil microorganisms play a crucial role in regulating the fertility of soil, health of plants, and cycling of C, N, and other nutrients^11,12^. Previous studies have shown that long-term continuous cropping can alter soil microbial communities^13–15^. In a continuous pea-cropping field, the soil microbial community was smaller, and the abundance of beneficial gram-positive bacteria and arbuscular mycorrhizal (AM) fungi was reduced^16^. Similarly, Sun *et al*. found that in continuous cropping of banana, bacterial community diversity continuously decreased and bacterial community composition and structure were affected^8^. Numerous studies have revealed that soil attributes are influenced by continuous cropping^17,18^. In addition, environmental factors, such as soil pH, affect soil microbial communities^19,20^. Wang *et al*. found that root exudate composition and soil pH affected soil microbial community in different plant growth stages^21^. Thus, it is crucial to analyse the relationship between soil microbial community and environmental factors. However, only a few studies have focused on differences in different areas under continuous cropping with the same plant.

Ramie, also known as “China grass,” is a perennial plant that belongs to the family Urticaceae. Ramie is a traditional fibre crop in China and an important natural fibre crop in India and other Southeast Asian and Pacific Rim countries. Owing to its high protein and amino acid content and rapid growth rate, ramie is used as a feed crop^22^. However, because of issues associated with continuous cropping, the cultivation areas of ramie have decreased sharply in recent years. Ramie is the main plant cultivated in the Hunan, Hubei, Sichuan, and Jiangxi Provinces of China. Because of differences in the climate and soil type, the rhizosphere microorganisms of continuous cropping ramie may change each year, thereby influencing the growth of ramie. Owing to the influence of different climatic conditions and soil types, the species and number of rhizosphere microorganisms in different planting areas of ramie deserve further study.

Crop rotation offers one way to solve continuous cropping problems; however, it is not applicable to ramie as it is a perennial crop. As soil microorganisms play an important role in plant health and crop yield, modulation of soil microbes provides an option for tackling continuous-cropping obstacles. In the early stage, we studied the changes in rhizosphere microorganisms in continuous cropping of ramie in the Hunan Province^3^. The main rhizosphere microorganisms negatively affecting the continuous cropping of ramie have been identified. However, microbial communities in ramie fields in different areas are poorly characterised. In this study, to obtain a comprehensive understanding of the bacterial and fungal community structures, we compared all continuous-cropping ramie fields located in four regions (Yuanjiang, Xianning, Sichuan, and Jiangxi) in China. To comparatively explore bacterial and fungal communities in different ramie planting areas, we subjected bacterial and fungal communities from continuous ramie cropping fields in Yuanjiang, Xianning, Sichuan, and Jiangxi (China) to high-throughput sequencing, and we used redundancy analysis (RDA) to analyse relationships between soil microbial communities and soil properties.

## Results

### Ramie-field soil basic properties

Basic properties of soils from continuous ramie cropping fields in Yuanjiang, Xianning, Sichuan, and Jiangxi are shown in Table 1. Soil pH ranged from 5.33 to 6.67. Soil pH was similar in Xianning and Jiangxi, and in Yuanjiang and Sichuan. Urease activity and TN were the highest in Yuanjiang, with values of 0.62 mg/kg/h and 1.52 g/kg respectively, and the lowest in Sichuan, with values of 0.43 mg/kg/h and 0.76 g/kg respectively. The highest available P level of 38.61 mg/kg was recorded in Sichuan and the lowest of 24.82 mg/kg in Jiangxi. Available K was the highest in Yuanjiang, with 162.60 mg/kg, and the lowest in Jiangxi, with 92.94 mg/kg (Table 1). Statistical analysis showed that the soil PH values of Xianning, Jiangxi, Yuanjiang, and Sichuan were significantly different and that there were significant differences between the total N (TN) and available K in all four regions (Table 1).

**Table 1:** The differences in soil chemical parameters in different areas of the continuous cropping ramie.

Parameters	yuanjiangR	xianningR	sichuanR	jiangxiR
PH	6.60 ± 0.057b	5.33 ± 0.088a	6.67 ± 0.088b	5.33 ± 0.081a
Urease (mg/k/h)	0.62 ± 0.010c	0.51 ± 0.008b	0.43 ± 0.015a	0.45 ± 0.007a
Total N (g/kg)	1.52 ± 0.03a	1.23 ± 0.063b	0.76 ± 0.063c	1.06 ± 0.003d
Available P (mg/kg)	29.89 ± 0.561b	25.66 ± 0.0.357a	38.61 ± 0.289c	24.82 ± 0.660a
Available K (mg/kg)	162.60 ± 1.438d	106.88 ± 0.284b	158.32 ± 1.351c	92.94 ± 0.741a
Soil T (°C)	16.50 ± 0.002	15.50 ± 0.001	13.50 ± 0.002	15.50 ± 0.001

Notes: the a, b, c and d indicate significant differences at p = 0.05.

### Overall diversity of microbial communities in ramie fields in different regions

The overall diversity of microbial communities is shown in Table 2. In total, 385,225 raw reads and 360,544 clean reads were obtained for bacteria, and 456,224 raw reads and 444,898 clean reads were obtained for fungi. The clean bacterial reads included 43,095 reads for Yuanjiang, 64,954 for Xianning, 110,987 for Sichuan, and 141,508 for Jiangxi. The clean fungal reads included 80,267 reads for Yuanjiang, 200,601 for Xianning, 95,671 for Sichuan, and 68,359 for Jiangxi. The α-diversities indicated that the microbial diversity was high in all soil samples. The observed bacterial species ranged from 546 to 2,606. The number of bacterial species was the highest in Sichuan and the lowest in Jiangxi. Observed fungal species ranged from 82 to 466. The number of fungal species was the highest in Xianning and the lowest in Jiangxi.

**Table 2:** Illumina Miseq reads, number of operational taxonomic units (OUTs), and alpha diversity of continuous cropping ramie in different areas.

Microbial community	Sample	Reads		Observed species	Alpha diversity		
		Raw	Clean		Shannon	Simpson	Chao1
Bacteria	yuanjiangR	48183a	43095a	2086b	8.5b	0.98b	3085b
	xianningR	67933a	64954a	2208b	8.4b	0.97b	3133b
	sichuanR	118874a,b	110987a,b	2606c	9.2b	0.98b	3758c
	JiangxiR	150235b	141508b	546a	3.7a	0.81a	872a
Fungi	yuanjiangR	84648a	80267a	371b	5.9c	0.93b	431b
	xianningR	201185b	200601b	466b	3.1a	0.67a	588c
	sichuanR	97696a	95671a	431b	6.4c	0.97b	475b
	jiangxiR	72695a	68359a	82a	4.2b	0.90b	112a

Notes: the a, b, c and d indicate significant differences at p = 0.05.

Moreover, the calculated bacterial and fungal α-diversity species richness (chao1), Simpson, and Shannon indices were all different in the four regions. For bacteria, the results of ANOVA of the Shannon, Simpson, and Chao1 diversity indices showed significant differences, with Jiangxi showing significantly lower diversity than other regions (Table 2). Similarly, for fungi, these indices showed significant differences between the four regions of continuous ramie cropping (Table 2).

### Soil microbial community composition in continuous cropping ramie fields in the different areas

**Figure 1:**
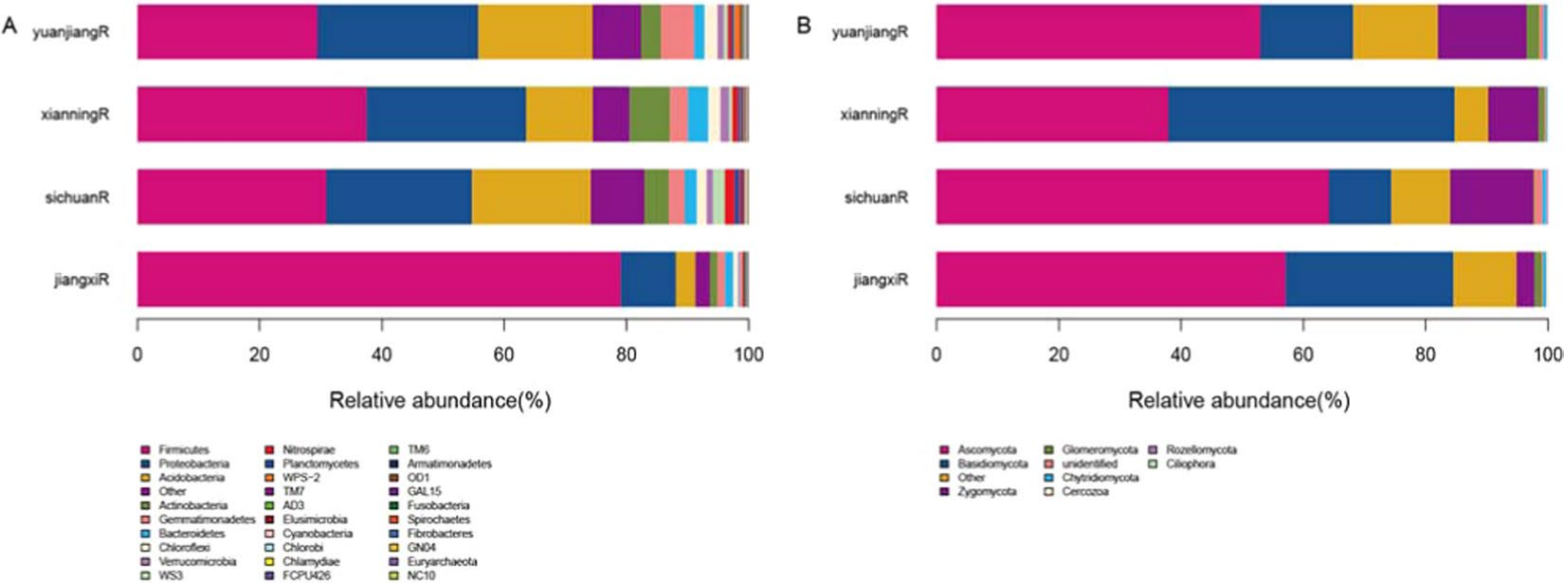
Relative abundance of the dominant bacterial (**A**) and fungal (**B**) taxa in four different area continuous cropping ramie soil samples at the phylum level. which were identified using the RDP classifier. Sequences not classified into any known group were designated as “other”.

Sequences that could not be classified into any known group were assigned as unclassified. The bacterial OTUs were assigned to 52 different phyla, 513 families, or 862 genera (Table S1). Five different phyla (Firmicutes, Proteobacteria, Acidobacteria, Other, and Gemmatimonadetes) out of the 52 total phylotypes were common to the four libraries, accounting for more than 85% of the total reads in each library (Fig. 1A, Table S1). Firmicutes was the most dominant phylum in all soil samples. In Jiangxi, this phylum accounted for 79.03% of all valid reads, which was more than that in other samples. Firmicutes accounted for 30.89%, 37.47%, and 29.46% of valid reads for Sichuan, Xianning, and Yuanjiang, respectively (P < 0.01, Table S2). Proteobacteria was the second dominant phylum. Compared with Firmicutes, Proteobacteria had a significantly lower abundance in Jiangxi (9.08%). In Xianning and Yuanjiang, Proteobacteria abundance was approximately 26% (P < 0.01, Table S2). Other bacteria, including Actinobacteria, Gemmatimonadetes, Bacteroidetes, Chloroflexi, Verrucomicrobia, WS3, Nitrospirae, Planctomycetes, TM7, WPS-2, AD3, Elusimicrobia, Cyanobacteria, Chlamydiae, Chlorobi, FCPU426, and TM6 were all the least abundant in Jiangxi.

We detected nine fungal phyla, with Ascomycota, Basidiomycota, and Zygomycota being the dominant (Fig. 1B). Ascomycota accounted for 37.96%–64.17% of all valid reads. Ascomycota members were the most abundant in Sichuan, with 64.17%, and the least abundant in Xianning, with 37.96%. Basidiomycota accounted for 10.25%, 15.23%, 27.44%, and 46.79% of the valid reads for Sichuan, Yuanjiang, Jiangxi, and Xianning, respectively (P < 0.05, Table S3). Zygomycota was the third dominant phylum in all samples, with an average relative abundance of 9.79% (P < 0.01, Table S3).

### Differences in microbial community among continuous-cropping ramie fields in different areas

**Figure 2:**
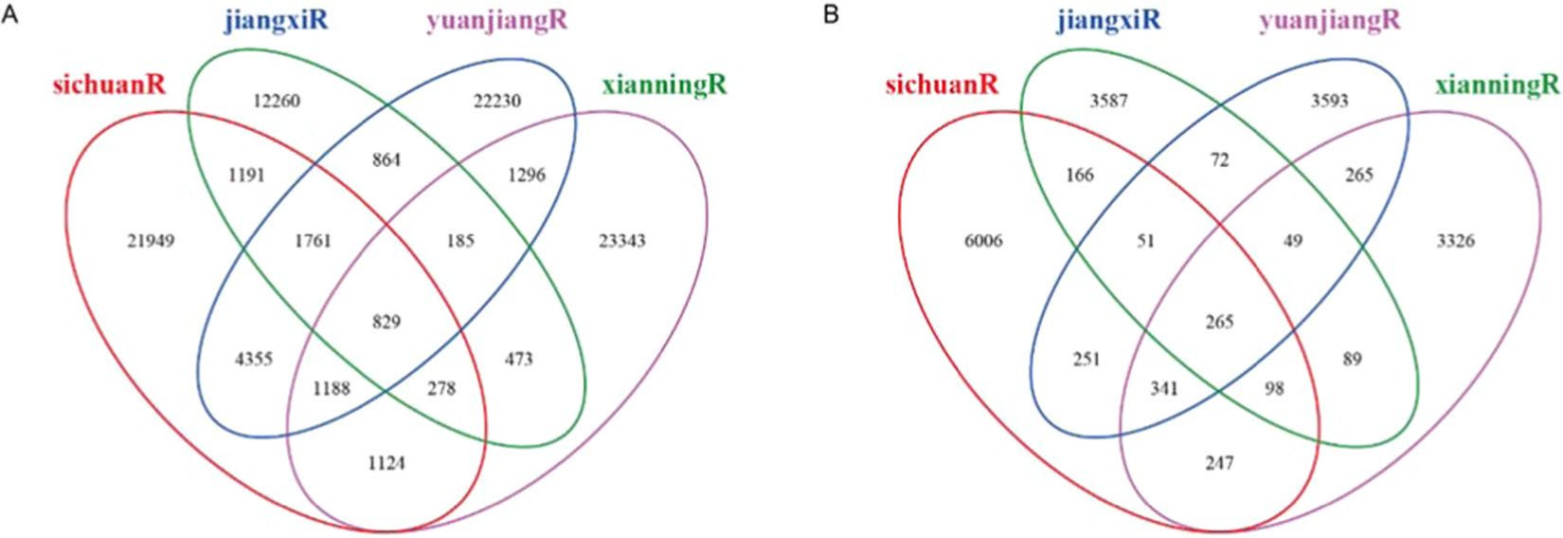
Venn diagram showing the number of unique bacterial (**A**) and fungal OTUs (**B**) detected in four different areas continuous cropping ramie soil samples.

To explore differences in microbial communities among all soil samples, Venn diagrams were generated based on OTUs, using Mothur. In total, 93,326 bacterial OTUs were detected in all samples, among which 21,949, 12,260, 22,230, and 23,343 OTUs were detected specifically in samples from Sichuan, Jiangxi, Yuanjiang, and Xianning, respectively, and 829 were shared by all samples (Fig. 2A). Totally, 18,406 fungal OTUs were detected, of which 6,006, 3,587, 3,593, and 3,326 were detected only in samples from Sichuan, Jiangxi, Yuanjiang, and Xianning, respectively, and 256 were shared by all samples (Fig. 2B).

**Figure 3:**
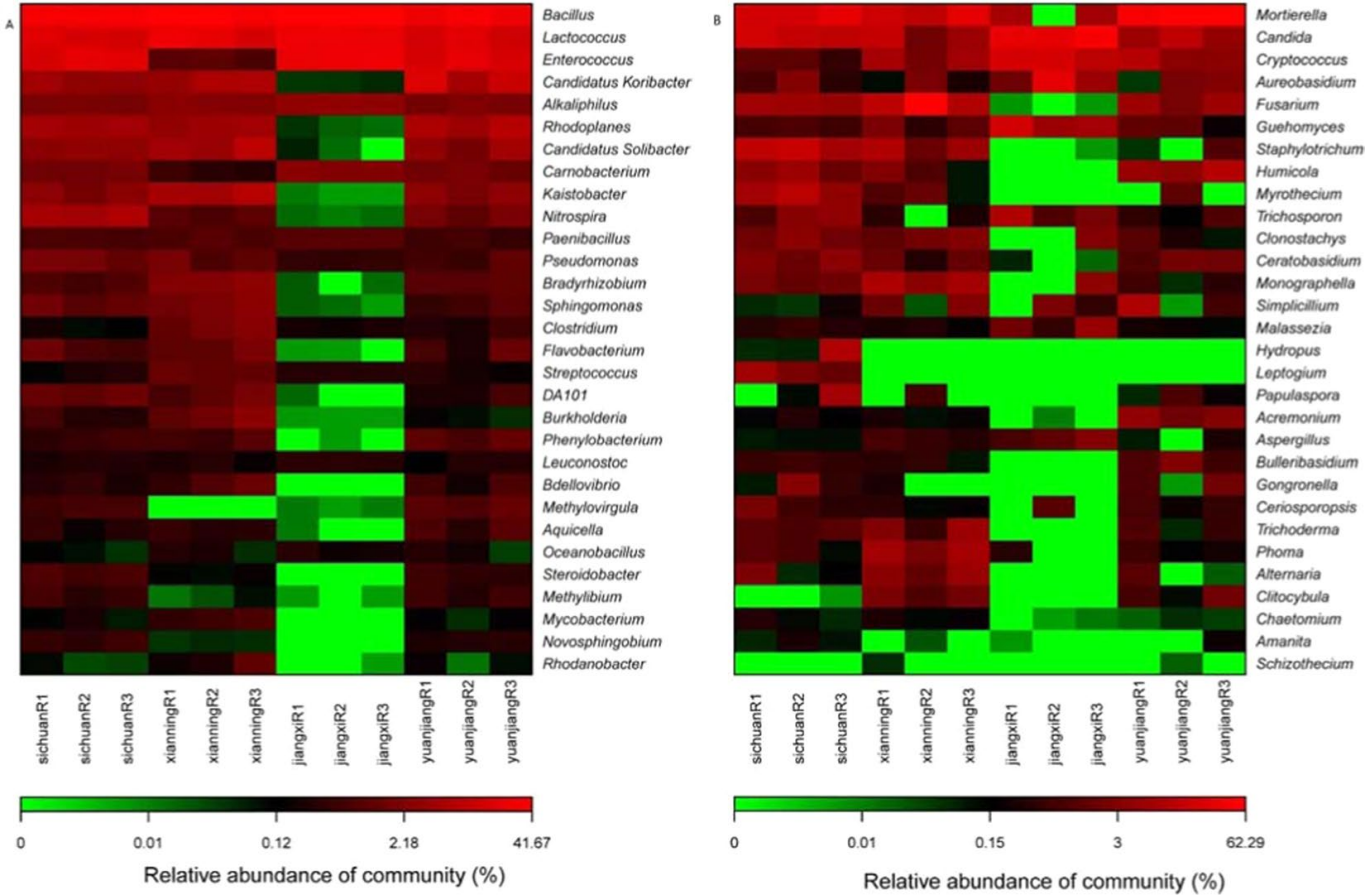
Heatmap analysis of bacterial (**A**) and fungi (**B**) based on the relative abundances of dominant genera from different areas continuous-cropping soil samples.

**Figure 4:**
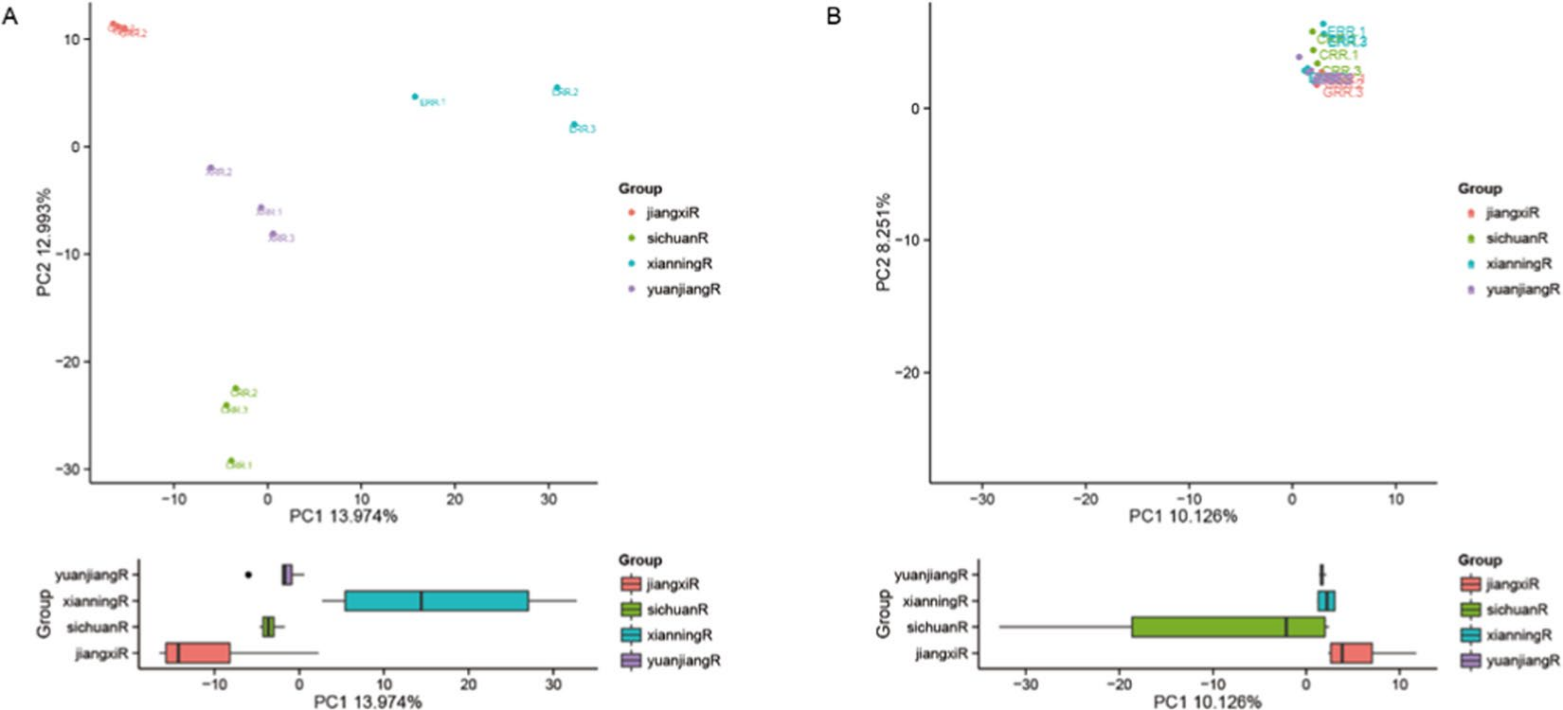
PCA of the OTUs detected major variations in the bacterial (**A**) and fungal (**B**) communities in four different area continuous cropping ramie soil samples.

The relative abundance of the 30 most dominant bacterial and fungal genera was visualised in heatmaps generated using custom R scripts. The bacteria *Bacillus* and *Lactococcus* presented a high relative abundance in all soil samples (Fig. 3A, P < 0.01, Table S2). *Enterococcus* was highly abundant in all samples, except in those from Xianning. Except for *Bacillus*, *Lactococcus*, *Enterococcus*, *Alkaliphilus*, and *Camobacterium*, other dominant genera had a low abundance (P < 0.01, Table S2). The fungi *Mortierella*, *Candida*, *Cryptococcus*, and *Aureobasidium* exhibited a high abundance in the four fields (Fig. 3B). *Fusarium* was highly abundant in Sichuan, Xianning, and Yuanjiang, but low in Jiangxi (P < 0.05, Table S3). Except for *Mortierella*, *Candida*, *Cryptococcus*, *Aureobasidium*, *Guehomyces*, *Trichosporon*, *Malassezia*, and *Aspergillus*, other genera presented a low abundance in Jiangxi. The heatmap showed that soil microbial composition was distinct in the different areas. The principal component analysis (PCA) was used to identify the community structure differences in different areas under continuous ramie cropping (Fig. 4). Two-dimensional plots of the coefficients of the first two principal components were generated to illustrate relationships among soil samples. As for bacteria, PC1 and PC2 contributed 13.974% and 12.993%, respectively (Fig. 4A). The PC1 value of Yuanjiang samples was similar to that of Sichuan samples, and Xianning samples had the highest PC1 value and Jiangxi samples had the lowest PC1 value. Although Jiangxi samples had the lowest PC1 value, the PC2 value was the highest. As for fungi, PC1 contributed 10.126% and PC2 contributed 8.25% (Fig. 4B). All soil samples had similar PC1 values. The PC2 values of the 12 samples were similar, but there were some differences; Xianning samples had the highest PC2 value and Jiangxi samples had the lowest PC2 value. In line with the heatmap results, the PCA results indicated that bacterial and fungal abundance differed among the four ramie-cropping areas.

### Correlation between community structure and environmental factors

**Figure 5:**
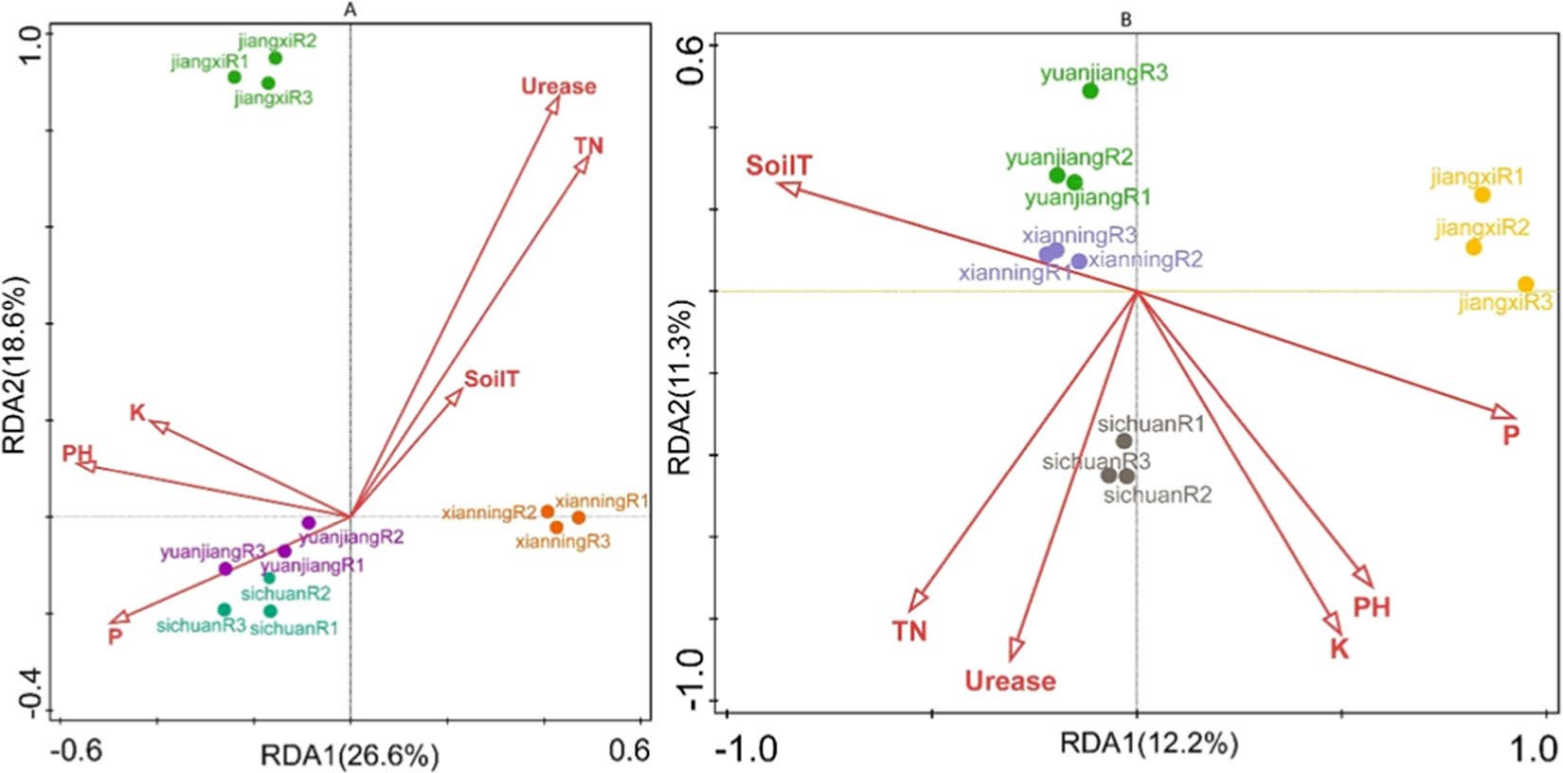
Redundancy analysis (RDA) based on bacterial (**A**) and fungal OUT (**B**) data with chemical parameters in four different area continuous cropping ramie soil.

In all samples, the bacterial OTUs were significantly positively correlated with the performance of several environmental factors; however, for fungi, only total T was significantly correlated with fungal community abundance (Table 3). To further identify the major environmental variables controlling the soil microbial community structure, the RDA was performed (Fig. 5A). The RDA based on OTU reads and all studied environmental variables was carried out for the continuous cropping ramie fields in the different regions. Relationships between bacterial communities and soil properties are shown in Fig. 5A (axis 1 = 26.6%, axis 2 = 18.6%), and relationships between soil properties and fungal communities are shown in Fig. 5B (axis 1 = 12.2%, axis 2 = 11.3%). The length of the arrow in the RDA plot indicates the degree of correlation between the environmental factor and sample distribution. The analysis revealed that the urease activity and TN exhibited the most significant correlation with bacterial community composition in all samples, whereas soil T was the least correlated with bacterial community composition in all soil samples (Fig. 5A, Table 3). The effects of urease activity, TN, and soil T were in the order Jiangxi > Xianning > Yuanjiang > Sichuan, and those of available K and P, and pH were in the order Sichuan > Yuanjiang > Jiangxi > Xianning. The effects of TN and P were in the order Sichuan > Jiangxi > Xianning > Yuanjiang (Fig. 5B). The RDA results demonstrated that environmental factors significantly affect soil microbial community composition.

**Table 3:** Relationship between environmental and number of operational taxonomic units (OUTs) of continuous cropping ramie in different areas.

OTUs	PH	Urease	TN	Available P	Available K	Soil T
Bacterial OTUs	P = 0.001**	P = 0.009**	P = 0.001**	P = 0.001**	P = 0.011*	P = 0.001**
Fungal OTUs	P = 0.162	P = 0.683	P = 0.001**	P = 0.012	P = 0.120	P = 0.068

Notes: * and ** indicate significant differences at p = 0.05 and p = 0.01, respectively.

## Discussion

In this study, using high-throughput sequencing, we analysed soil bacterial and fungal communities in continuous-cropping ramie fields in Yuanjiang, Xianning, Sichuan, and Jiangxi. According to the α-diversity analysis, the overall diversity of bacterial and fungal community compositions differed among the soil samples. For bacteria, the Shannon, Simpson and Chao1 α-diversity indices revealed that the diversity of Jiangxi was significantly lower than that of the other three regions (Table 2). This may be due to different soil properties and climactic conditions in the different regions. For bacteria, Firmicutes, Proteobacteria, and Acidobacteria were the dominant phyla, which was in accordance with the findings of our previous study^3^. These phyla were also dominant in continuous-cropping fields of tobacco^6^, peanut^15^, and soybean^23^. Proteobacteria members play key roles in C, S, and N cycling in soil^24^. In healthy soil with adequate fertilisation, the abundance of Proteobacteria increases, whereas it decreases when nutrients are exhausted^21^. However, in our study, the percentage of the dominant phyla was different among the four regions under continuous ramie cropping. The abundance of Firmicutes was the highest in Jiangxi, accounting for 79.03% of all valid reads, which was higher than that in other samples (Fig. 1A). Data from numerous studies show that most rhizosphere species belong to the phylum Firmicutes^25–27^. It may be that the proportion of *Firmicutes* is so large that the α-diversity index in the previous analysis in Jiangxi was significantly lower than that in the other three places. The increase in Firmicutes may also be one of the main barriers to the continuous cropping of ramie in Jiangxi; this point needs further investigation. *Bacillus*, *Lactococcus*, *Koribacter*, *Enterococcus*, and *Alkaliphilus* were the dominant bacterial genera, and *Bacillus* was the most dominant genus in all samples. *Bacillus*, which belongs to Firmicutes, can promote plant growth and control soil-borne diseases as a beneficial microbe^28^. For example, *Bacillus* restrains bacterial wilt caused by *Ralstonia solanacearum*^29–32^. Furthermore, *Bacillus*-inoculated fertiliser reportedly increases soil bacterial diversity^33^. Hence, *Bacillus* is a good choice for improving the soil microbial community. Interestingly, except for *Bacillus*, *Lactococcus*, *Enterococcus*, *Alkaliphilus*, *Camobacterium*, and *Paenibacillus*, the abundance of 24 other genera were lower in Jiangxi. The decrease in these species directly led to the decrease in rhizosphere microbial diversity in Jiangxi. Ascomycota, Basidiomycota, and Zygomycota were the dominant fungal phyla, which was consistent with the findings of previous studies in citrus^34^ Ascomycota and Basidiomycota are important fungi in most soils^35,36^, and the species in both phyla are involved in C cycling by degrading organic substances^37,38^. We observed significant differences in the relative abundance of *Ascomycota* and *Basidiomycota* in our samples; especially in Sichuan, the abundance of *Ascomycota* was the highest compared with that in the other three regions. The RDA results revealed that the urease activity and TN had the lowest effects in Sichuan. Thus, we speculate that under the condition of low TN in Sichuan, Ascomycota may participate in C cycling by degrading organic substances.

Soil properties, including soil pH and available N, are influenced by continuous cropping^18,39^. Furthermore, soil microbial communities are affected by environmental factors, including soil pH, TN, and T^40–43^. Hence, we used the RDA to analyse relationships between soil microbial composition and environmental factors (including soil pH, soil T, available P, available K, TN, and urease activity). The analysis revealed that different environmental factors differentially affected the bacterial and fungal communities. Furthermore, we carried out the correlation analysis between environmental and number of operational taxonomic units (OTUs) of continuous cropping ramie in the different areas (Table 3). All six environmental factors were significantly correlated with bacterial communities; however, there was no significant correlation between fungi and any of the tested parameters, except with TN (Table 3). Numerous studies have found environmental factors to differentially affect bacterial and fungal communities^44–46^. However, in this study, environmental factors were found to have a greater effect on bacterial diversity than on fungal diversity. The urease activity was the most significantly correlated with bacterial and fungal communities (Fig. 5). Urease catalyses the breakdown of urea into CO_2_ and NH_3_, and might be a good index of soil quality^47,48^. Bacteria as well as plants secrete urease^49^. Continuous cropping causes a decline in the urease activity, and significant correlations between urease activity and bacterial networks have been previously found^50,51^. We found that TN was significantly correlated with microbial communities in all samples (Fig. 5). Previous studies have demonstrated that N primarily regulates the bacterial community^52,53^. Lei *et al*. found that the urease activity increased when the N application rate was increased from 247 to 433 mg/kg^54^. Liang *et al*. reported that a medium N level increased the urease activity^55^. Thus, N fertilisation provides a good means to increase the urease activity to improve the soil microbiota. However, high quantities of ammonia reduce the urease activity^56^; therefore, TN is a factor that has the greatest influence on the diversity of rhizosphere bacteria and fungi. The N fertilisation dose is crucial in different regions of continuous cropping ramie.

In conclusion, our study indicated that microbial community diversity and composition in continuous-cropping ramie fields differed among Yuanjiang, Xianning, Sichuan, and Jiangxi. However, some common features also exist; Firmicutes, Proteobacteria, and Acidobacteria were the dominant bacterial phyla, accounting for more than 85% of the total reads in each area, and Ascomycota, Basidiomycota, and Zygomycota were the dominant fungal phyla. Furthermore, environmental factors, including the urease activity, TN, and soil T, affected the microbial communities. However, based on our findings, the effect of soil environmental factors on the bacterial diversity of continuous cropping ramie was greater than that on fungal diversity. According to the correlation analysis of root microorganisms and environmental factors in several different places, the TN and urease in soil were the key factors influencing microbial diversity and thus the main targets to solve the problem of continuous cropping and the growth of ramie. We suggest that regulating the microbial community by modulating the urease activity and TN content might provide a means to tackle problems caused by continuous cropping.

## Methods

### Site description and sample collection

The experimental sites were located in Yuanjiang (Hunan Province, 112°38′84.35″N, 28°83′76.29″E), Xianning (Hubei Province, 114°25′21.29″N, 29°91′10.33″E), Sichuan (Sichuan Province, 107°51′84.07″N, 31°22′25.1″E), and Jiangxi (Jiangxi Province, 114°42′15.3″N, 27°78′83.96″E). All soil samples were collected in May 2016 from four fields with more than eight years of continuous ramie cropping. May is the season of vigorous ramie growth and the period when ramie rhizosphere microorganisms are highly active. Therefore, sampling at this time is representative. In each study area, the variety of ramie cultured is Zhongzhu no.1. Ramie plants with similar growth in four different planting areas were selected as sampling objects. We collected soil samples close to the root systems of five plants and pooled them into one sample. From each area, we collected three pooled samples, which were labelled as the province name followed by “R1,” “R2,” and “R3” (e.g., for the Yuanjiang area, samples were named yuanjiangR1, yuanjiangR2, and yuanjiangR3). Soil samples were sifted through a 2-mm sieve and homogeneously mixed. Twelve soil samples were stored in plastic bags and transferred on ice to the laboratory. One half of each soil sample was stored at −70 °C for biological and biochemical analyses and the other was air-dried at room temperature for one week for chemical analysis. Each sample was analysed in triplicate.

### Analysis of soil basic properties

Soil basic properties, including pH, TN, available P, available K, soil temperature (Soil T), and urease activity were analysed. Basic chemical properties were analysed according to published procedures^34^, soil pH was measured in a 1:5 (w/w) soil:CO_2_-free distilled water suspension. TN was determined using the Kjeldahl method. Available P was determined by molybdenum antimony blue colorimetry after digesting the sample with a mixture of perchloric acid and sulphuric acid. The Olsen method was used to measure available K. Soil T was monitored using a portable probe attached to the Li-8100 system. Soil urease activity was determined by an improved sodium phenate and sodium hypochlorite colorimetric method^57^.

### DNA extraction

The total genomic DNA was extracted from the soil samples using the E.N.Z.A. Soil DNA Kit (Omega Bio-tek), following the manufacturer’s protocol (http://omegabiotek.com/store/product/soil-dna-kit/). DNA quantity and quality were evaluated by spectrophotometry (NanoDrop) and agarose gel electrophoresis, respectively. The DNA was diluted to 1 ng/μL and stored at −20 °C until further analysis.

### PCR amplification and Illumina high-throughput sequencing analysis

Bacterial 16S and fungal ITS rRNA gene sequences were amplified using barcoded primers and HiFi Hot Start Ready Mix (Kapa Biosystems). For bacterial diversity analysis, we used the primers 338F (5′-ACTCCTACGGGAGGCAGCAG-3′) and 806R (5′-GGACTACHVGGGTWTCTAAT-3′), which amplify the flexible V3-V4 regions of the 16S rRNA gene. For fungal diversity analysis, we used the primers fITS7 (5′-GTGARTCATCGAATCTTTG-3′) and ITS4 (5′-TCCTCCGCTTATTGATATGC-3′), which amplify the ITS2 region. PCR amplicon quality was evaluated by gel electrophoresis, and the amplicons were purified using AMPure XP beads (Agencourt). The amplicons were then amplified in a second round of PCR, subjected to purification using AMPure XP beads, and quantified using the Qubit dsDNA Assay Kit. Equimolar amounts of purified amplicons were pooled for sequencing. Amplicons were subjected to high-throughput sequencing on an Illumina Mi-Seq platform (Illumina, San Diego, CA, USA) at OE Biotech (Shanghai, China). All sequence data have been deposited in the NCBI Sequence Read Archive database under accession number PRJNA543166. After raw paired-end reads were quality-filtered with Trimmomatic software, FLASH software was used for paired-end read assembly^58^. QIIME software (version 1.8.0) and UPARSE pipeline were applied to analyse the sequences^59^. Then, UPARSE pipeline was used to explore OTUs at 97% similarity^60^ Using a representative sequence of each OTU, taxonomic composition as a.ssigned using the RDP classifier^61^.

### Data analyses

For all parameters tested, multiple comparisons were conducted using the one-way analysis of variance followed by Tukey’s honest significant difference multiple-range tests. For α-diversity, all analyses were based on OTU clusters, with a cut-off of 3% dissimilarity. The Chao1 index was calculated to estimate the richness in each sample. Diversity within each sample was estimated using the nonparametric Shannon diversity index. Rarefaction curves based on the average number of observed OTUs were generated using Mothur software to compare the relative levels of bacterial and fungal OTU diversity across continuous-cropping ramie field soil samples. For β-diversity, hierarchical cluster dendrograms (with Bray–Curtis distance dissimilarities) were generated using Mothur, based on the OTU composition. The dendrograms were used to compare bacterial and fungal community structures among all soil samples. Heatmaps and Venn diagrams were generated using custom R scripts. Weighted and unweighted UniFrac distance metrics (based on the phylogenetic structure) were used to generate principle coordinate analysis (PCA) plots to assess similarities in community composition among the different samples. The RDA was used to study relationships between bacterial and fungal communities and soil properties. Histograms were created in SPSS and Microsoft Excel 2010.

## Supplementary information

Supplementary Information1.

Supplementary Information2.

Supplementary Information3.
